# Alpine Diet in Valmalenco (Lombardy, Italy): Nutritional Features of Spontaneous Plants and Traditional Dishes

**DOI:** 10.3390/nu15081988

**Published:** 2023-04-20

**Authors:** Fabrizia Milani, Martina Bottoni, Claudia Giuliani, Lorenzo Colombo, Maria Cristina Casiraghi, Paola Sira Colombo, Piero Bruschi, Daniela Erba, Gelsomina Fico

**Affiliations:** 1 Department of Pharmaceutical Science, University of Milan, 20133 Milan, Italy; fabrizia.milani@unimi.it (F.M.); martina.bottoni@unimi.it (M.B.); claudia.giuliani@unimi.it (C.G.); lorecolo.93@gmail.com (L.C.); pasico19@virgilio.it (P.S.C.); gelsomina.fico@unimi.it (G.F.); 2 Botanical Garden G.E. Ghirardi, Department of Pharmaceutical Science, University of Milan, 25088 Toscolano Maderno, Italy; 3 Department of Food, Environmental and Nutritional Sciences DEFENS, University of Milan, 20133 Milan, Italy; maria.casiraghi@unimi.it; 4 Department of Agricultural, Environmental, Food and Forestry Science and Technology, University of Florence, 50144 Florence, Italy; piero.bruschi@unifi.it

**Keywords:** ethnobotany, Alpine diet, carotenoids, phenolic compounds, digestible starch, spontaneous plants, traditional dishes, gnocchi

## Abstract

Background: Along the Alps, the Alpine diet is considered to be one of the most common nutritional models. Next to traditional animal-based products, spontaneous plants of the territory are collected and eaten. Aim: The aim of this study is to evaluate the nutritional features of selected autochthonous plants of the territory and the typical recipe of green gnocchi. Methods: The analyses of proximate composition, carotenoid, total phenol, and mineral contents in raw and cooked plant samples and the chemical composition and in vitro starch digestibility in green and control gnocchi were performed. Results: Except for *Aruncus dioicus,* all the wild plants contained high levels of carotenoids (15–20 mg/100 g FW), mainly as xanthophylls. *Rumex acetosa* showed the highest levels of total phenols (554 mg GAE/100 g FW), and *Urtica dioica* can be considered to be a good dietary source of iron, calcium, and magnesium (4.9, 410, and 72 mg/100 g FW). Cooking significantly decreased the potassium and magnesium contents in all wild species, and total phenols and carotenoids in *Aruncus dioicus, Blitum bonus-henricus*, and *Silene vulgaris* (*p* < 0.05). The slowly digestible fraction of starch (%SDS/available starch), which is inversely correlated to insulin demand, was significantly increased in green gnocchi compared to matched control gnocchi (*p* < 0.05). Conclusions: Traditional consumption of spontaneous plants in the Alpine regions might increase the dietary intakes of several bioactive substances and contribute to cover the nutritional needs of micronutrients.

## 1. Introduction

Italy has always been considered to be the homeland for the Mediterranean diet, which is a nutritional model theorized by the American physiologist Ancel Keys in the 1950s and 1960s of the 20th century [1]. According to Naska and Trichopoulou (2014), the Mediterranen diet is characterized by an abundant consumption of plant-based foods and fresh fruits, whereas animal-based products, such as fish, poultry, and eggs are eaten in low to moderate amounts. Red meat is rarely consumed. Additionally, olive oil is the principal fat, used raw or in the preparation of dishes, while wine is consumed in low to moderate amounts [2]. Since its theorization, this dietary pattern has been widely studied, specifically as a potential tool for the primary and secondary prevention of several types of diet-related pathologies, such as cardiovascular diseases, metabolic syndrome, and cancers [3,4,5]. However, interest in the Mediterranean diet is not only related to its health properties. In 2013, the UNESCO included it in the Intangible Cultural Heritage of Humanity list since it “emphasizes values of hospitality, neighborliness, intercultural dialogue, and creativity, and a way of life guided by respect for diversity. It plays a vital role in cultural spaces, festivals, and celebrations, bringing together people of all ages, conditions, and social classes” [6].

In addition, Italy is also characterized by the presence of mountainous spans, which, since ancient times, have connoted the culinary culture of some areas with their territorial and pedoclimatic peculiarities. Specifically, the Alps cross the northern part of Italy and are considered to be the most important European mountain range. The dietary pattern adopted throughout the Italian Alpine regions deviates from the Mediterranean diet with respect to food groups (i.e., consumption of fish is limited and mainly related to freshwater), typical local products, and traditions. The Alpine diet may be considered to be the nutritional model in these regions, with a pattern rich in animal-based agropastoral products (i.e., milk, butter, cream, and cheese from cows, goats, and sheep; fresh and cured meats; corn polenta, mixed-grain flours; bread and pasta; or alternative flours such as buckwheat and chestnut). Interest in the Alpine diet, its traditions, and products has been such that, in 2018, the application procedure for its recognition in the Intangible Cultural Heritage of Humanity list was initiated by the AlpFoodway project, within the framework of the Interreg Alpine Space Programme [7,8].

Although the dietary pattern of the Alpine diet is mainly known for its animal-based products, which could pose a problem concerning their health value (i.e., due to the high content of saturated fatty acids, cholesterol, sodium, etc.), the Alpine diet is also characterized by the collection and consumption of seasonal spontaneous plant species, unprocessed or in local traditional dishes [9,10,11,12,13,14,15,16,17]. Despite the importance and spread of this dietary model in the Alpine regions, very few epidemiological studies have been conducted in this area [18], as well as little can be found in the scientific literature regarding the nutritional analysis of its typical ingredients and products [19]. Moreover, while the scientific literature is rich in information regarding wild plants collected in the Mediterranean regions [20,21,22], to the best of our knowledge, there have been no nutritional evaluations on spontaneous plant species collected in the studied area, nor on plant-based traditional dishes. Although, undoubtedly, the Mediterranean and the Alpine areas share many species, nutritional analyses on plants grown in completely different conditions would be of extreme interest [23].

The work presented herein stemmed from an ethnobotanical survey conducted on the Alpine region of Valmalenco (Sondrio, Lombardy, Italy), which has never been investigated before, in the context of the wider European Italy-Switzerland Interreg B-ICE research program (2019–2023) [24]. This project is aimed at creating a new management model for the ongoing climate crisis and new opportunities of territory enhancement for tourism attraction. Valmalenco is an Alpine valley surrounded by the Bernina Alps and it includes five municipalities: Caspoggio (1098 m a.s.l.), Chiesa in Valmalenco (800 m a.s.l.), Lanzada (1000 m a.s.l.), Spriana (754 m a.s.l.), and Torre di S. Maria (795 m a.s.l.). Given the relative isolation of the valley from major urban centers and the rich plant biodiversity that characterizes the territory, the people of Valmalenco still use autochthonous spontaneous species daily in several aspects of their lives [25].

Specifically, during our investigation in this area, we collected information concerning the traditional use of plants in the food sector. All data gathered over the course of the three-year field work allowed us to select plant species for the evaluation of the following nutritional features: (a) content in macro- and micronutrients in raw and cooked samples (b) content in bioactive compounds (specifically, carotenoids and polyphenols) in raw and cooked samples; (c) starch availability, in traditional dishes.

Following our previous study [25], the objective of this work, therefore, was to deepen the knowledge concerning the traditional uses of the plant species in the area, with special attention given to the nutritional value of spontaneous plant species that are an integral part of the typical Alpine diet of the Valmalenco territory.

## 2. Materials and Methods

### 2.1. Ethnobotanical Investigation, Data Archiving, and Processing

The ethnobotanical investigation and the data archiving and processing followed Bottoni et al. (2020) [25]. The Ethics Committee of the Università degli Studi di Milano approved the study protocol (Protocol number 40/23).

Concerning the selection of the species that were to be subsequently analyzed, all the fruits and berries that were mainly used for jams and desserts were ruled out due to the high content of free sugars in the preparations. Then, species used in alcoholic beverages or as mere flavorings for traditional dishes or grappa were excluded. Thus, special attention was given to the spontaneous plants that were either eaten cooked as a vegetable side dish or as a main ingredient of a typical preparation of Valmalenco, i.e., the gnocchi. Particular interest was also piqued by the direct consumption as field snack of some of the species.

Therefore, the final selection included *Aruncus dioicus* (Walter) Fernald (sprouts, raw and cooked) (AS); *Blitum bonus-henricus* L. (young leaves, raw and cooked; gnocchi) (BB); *Rumex acetosa* L. (young leaves, raw) (RA); *Silene vulgaris* (Moench) Garcke (young leaves, raw and cooked) (SI); *Taraxacum officinale* Weber (young leaves, flowers and young roots mix, raw; leaves, raw and cooked) (TA); and *Urtica dioica* L. (young leaves, raw and cooked; gnocchi) (UR).

For these 6 species, *exsiccata* were collected, conveniently coded, and preserved at the Herbarium of G.E. Ghirardi Botanical Garden (DISFARM, University of Milan).

### 2.2. Plant Collection and Processing

Plant material was collected between May and June 2021. For each of the 6 plant species, at least two different gatherers were selected among the informants, based on their knowledge of the territory traditions and their availability. Then, each gatherer was associated with a species and given a univocal identification code. For example, we cite the codes RA.1 and RA.2 for the gatherers of fresh leaves of *Rumex acetosa* L.

Then, each species was collected in the places where gatherers typically harvested them for their own consumption. There were two different collections of the same species which were performed during the same day whenever possible. The same plant parts were collected by two different informants, with some exceptions due to discrepancies in the traditional uses in different parts of the valley. For this reason, all information was noted and considered during the subsequent laboratory analyses. Finally, all data about collection time, altitude, temperature, and humidity were filed away.

Then, parts of the collected plants were washed and boiled in tap water by the informants, with the supervision of two members of the research group. Thus, for each species, we obtained raw and boiled samples, with the exception of the leaves of *R. acetosa*, and the leaves, flowers, and root mix of *Taraxacum officinale* Weber, which are only consumed raw throughout the territory.

Then, every sample was refrigerated or frozen at −20 °C, transported back to the laboratory within 24 h from the collection, maintaining the cold chain, and quick-frozen at −80 °C, before freeze-drying. All data about the collection and processing steps are summarized in Table 1.

### 2.3. Traditional Culinary Preparations

For the species *Blitum bonus-henricus* L. and *Urtica dioica* L., in addition to the raw and cooked samples, a traditional dish of Valmalenco, namely gnocchi or green gnocchi, was prepared. Specifically, two gatherers for each species prepared a quantity of gnocchi suitable for the subsequent laboratory analyses, with the supervision of two members of the research group. The dough, containing blended cooked leaves of nettle or wild spinach, was made following the family recipes of the informants, carefully noted and reported in Table 2, divided in gnocchi with a spoon, and blanched in salted boiling tap water for 5 min. For each batch of gnocchi, a corresponding control batch was prepared and blanched, following the original recipe and containing all the ingredients except for the plant, which was substituted with water. Then, the gnocchi were frozen at −20 °C and transported back to the laboratory within 24 h from the preparation, maintaining the cold chain.

### 2.4. Chemicals

The carotenoid standards were lutein, zeaxantin, violaxantin, and β-carotene (CaroteNature, Münsingen, Switzerland). Gallic acid (842649), pepsin (P7000; ≥250 U/mg), pancreatin (P7545; 8xUSP), invertase (I4504; ≥300 U/mg), amyloglucosidase (A7095; ≥260 U/mL), Folin–Ciocalteu phenol reagent (109001), nitric acid 60% ultrapure (101518), and chemicals, at analytical or LiChrosolv grade, were purchased from Merck KGaA (Darmstadt, Germany).

### 2.5. Chemical Composition

The moisture analyses were performed on fresh and freeze-dried samples, and proximate composition analyses were performed on raw freeze-dried samples, according to the approved methods of the American Association of Cereal Chemists (AACC) methods: 44-15.02 moisture, 46-12.01 crude protein, 30-10.01 crude lipid, and 08-01.01 ash [26]. The free sugars were analyzed by HPLC, according to Rocklin and Pohl (1983) [27] and the total starch content was analyzed using a kit (K-TSTA, Megazyme, Wicklow, Ireland), and their sum was expressed as available carbohydrates. Not-available carbohydrate contents were determined by calculating the percent remaining after all the other components were measured. The proximate analysis was also performed in cooked gnocchi samples (with and without plants), together with the analysis of resistant starch by using enzymatic kits (K-RSTAR, Megazyme, Ireland).

### 2.6. Nutritional Value

The analyses of carotenoids on freeze-dried samples were performed by HPLC on raw samples in order to characterize the carotenoid compounds, according to Granado et al. (2001) [28]. Briefly, freeze-dried samples were extracted with a mix of hexane/methanol/tetrahydrofuran (2:1:1) until a colorless extraction solution was obtained. Residues, obtained by vacuum evaporation at low temperature, were solved in absolute ethanol/tetrahydrofuran (21:4), filtered (PTFE) and injected into the HPLC system consisting of an Alliance (mod. 2695 Waters, Milford, MI, USA) equipped with a DAD detector column (mod. 2996 Waters) and a Develosil RP-Aqueous (250 × 4.6 mm) (Phenomenex, Torrance, CA, USA). A mixture of methanol-tetrahydrofuran-water (89:7:4 *v*/*v*), as mobile phase with a flow rate of 1.0 mL/min, was applied. The quantification of carotenoids was performed in relation to standard curves of carotenoids. The results are expressed as mg of carotenoid/100 g fresh weight (FW). The contents of retinol equivalent (RE) were calculated on the basis of β-carotene (mg β-carotene/6 × 1000 = µg RE/100 g FW).

A colorimetric method [29] was applied to determine total carotenoid contents in raw and cooked samples. An aliquot of freeze-dried samples was extracted with ethanol/tetrahydrofuran (21:4), vortexed, and dark incubated at room temperature (1 h). Filtered supernatants were analyzed in a UV-Vis spectrophotometer (Evolution 201, Thermo Scientific, Lenexa, KS, USA) at 665 nm, 649 nm, and 470 nm, against a solvent mixture. Total carotenoid (carotenes and xanthophylls) amounts were assessed following the equation proposed by the method and expressed as mg total carotenoids/100 g FW.

Total phenols were quantified spectrophotometrically through the Folin–Ciocalteu test on raw and cooked species [30]. An aliquot of samples was extracted for 2 h with methanol 80% under stirring, twice [31]. The phenol contents were quantified based on a standard calibration curve of gallic acid and expressed as mg of gallic acid equivalents (GAE)/100 g FW.

For the determination of elements of interest (Mg, K, Ca, P, Mn, Fe, Cu, and Zn), freeze-dried raw and cooked samples were exactly weighted (0.2 g), and then digested using a microwave digestion system (MULTIWAVE-ECO, Anton Paar, Rivoli, Italy) in Teflon tubes filled with 10 mL of 65% HNO_3_ by applying the following a one-step temperature ramp: the temperature was increased to 210 °C in 10 min and maintained for 10 min. After cooling, the mineralized samples were transferred in polypropylene test tubes. The concentration of elements was measured by ICP-MS (Aurora-M90 ICP-MS Bruker Daltonik GmbH, Leipzig, Germany), quantified by means of standard solutions, and expressed as mg/100 g FW.

Cooked gnocchi, with or without vegetable species, were thawed with a quick pass in boiling water, and then ground before the analysis of in vitro starch digestibility [32]. The method measures the rate of digestion through a series of proteolytic and amylolytic enzymatic attacks under controlled conditions. Based on the HPLC analysis of the glucose released at the specific time of hydrolysis, the fractions of rapidly (RDS) and slowly (SDS) digestible starch were calculated. The sum of RDS and SDS, defined as available starch (AvSt), is indicative of the starch digestible in the small intestine. The results are expressed as the percentages of RDS and SDS over AvSt. For each gnocchi sample, the digestibility tests were conducted in duplicate, repeated three times.

### 2.7. Statistical Analysis

A statistical analysis was performed on the proximate and nutritional variables by means of the STATA software (StataCorp, version 12.0, College Station, TX, USA). Data were elaborated using the Kruskal–Wallis test in order to assess the influence of botanical species on nutrient contents. An analysis of the difference between species was assessed by Dunn’s test with *p* < 0.05 as the level of statistical significance. The comparison between control and green gnocchi was performed using the Mann–Whitney test with *p* < 0.05 as the level of statistical significance. The relationship between total carotenoid contents measured by the two analytic methods was assessed by Pearson’s correlation coefficient. All analyses were performed in triplicate.

## 3. Results and Discussion

### 3.1. Ethnobotanical Survey and Species Selection

A total of 401 informants, aged between 11 and 97, were interviewed throughout the territory of Valmalenco between June 2019 and April 2022. Among these informants, 396 informants reported information concerning the food sector, for a total of 6.969 citations. The survey provided data on 154 plant species, 132 of which spontaneous, belonging to 54 botanical families. The most cited families were Rosaceae (no. of citations = 1638 and no. of species = 20), Asteraceae (no. of citations 1147 and no. of species 20), Ericaceae (no. of citations 1102 and no. of species 3), and Lamiaceae (no. of citations 503 and no of species 19). By selecting only data concerning first person and ongoing usage, as recounted by the informants, the most cited species were *Vaccinium myrtillus* L. (no. of citations = 709), *Rubus idaeus* L. (no. of citations = 510), and *Taraxacum officinale* Weber (no. of citations = 453). Table 3 shows the species that were cited at least 50 times by the people of Valmalenco.

Specifically, *V. myrtillus*, *R. idaeus*, *V. vitis-idaea*, *F. vesca*, *R. ulmifolius*, and *R. rubrum* were mostly consumed as fresh fruits, jams, or as ingredients for the preparation of cakes and desserts. A mix of fresh leaves, flowers, and roots of *T. officinale* was generally eaten raw, in spring, to accompany hard-boiled eggs, while the flowers were used for the preparation of the so-called dandelion ”honey” (a thick sugary syrup made with dandelion flowerheads, sugar, water, and lemon juice). Sometimes, in late spring, the older and more bitter leaves were boiled before eating. A syrup of flowers of *S. nigra* was usually diluted in water for a refreshing drink, while its fruits were cooked with sugar for hours to obtain a tart and sweet jam. The flowerheads of *A. genipi* and *A. moschata*, as well as the hypogeal parts of *G. lutea* and the green pine cones of *P. mugo*, are known to have been the key ingredients of some of the traditional alcoholic bitters of the valley or were used for flavoring grappa. Young leaves of *U. dioica*, *B. bonus-henricus* (wild spinach), *S. vulgaris*, and *M. sylvestris* and the sprouts of *A. dioicus* (called wild asparagus) and *H. lupulus* were eaten, boiled as a side of vegetables, or added as ingredients for traditional dishes such as gnocchi, pastas, risottos, and soups. The leaves of *Thymus spp*. and *S. officinalis* and the galbuli of *J. communis* were used as flavors for different types of traditional dishes, which included venison and sauces, or for grappa. The black juniper berries were also mentioned as a snack, directly eaten in the fields. Despite some difficulties in the collection and processing, the false fruits of *R. canina* and the fruits of *P. spinosa* were often cited for the preparation of jams or eaten as a field snack. Rose hips and flowers, fresh or dried, were also blended in pleasant herbal teas. Finally, fresh leaves and young stems of *R. acetosa* were eaten raw, directly in the field, as a thirst-quenching snack, due to their acidulous taste.

### 3.2. Chemical Composition

The results of the proximate composition analyses are reported in Table 4 for raw vegetables and in Table 5 for gnocchi. Data of vegetable species are reported as the median (25–75° percentile) of the results for the two harvests of *U. dioica* (UR), *R. acetosa* (RA), *S. vulgaris* (SI), *A. dioicus* (AS), and B. *bonus-henricus* (BB) and of the four harvests of *T. officinale* (TA) (the two samples of leaves and the two samples of a mix of leaves, flowerheads, and root). Data of green and control gnocchi are reported for each culinary preparation due to the difference in traditional recipes (Table 5).

Limited data of proximate composition of the wild vegetables considered in the present study could be found in the literature. García-Herrera et al. (2020) reported the results of the proximate analysis of *S. vulgaris* in their study on the nutritional and phytochemical composition of wild edible Mediterranean plants [20], and Tardio et al. reported the chemical composition of RA, SI, and TA [33]. On the contrary, more studies have focused on chemical composition of *U. dioica* [33,34,35,36]. The latter study, performed on two successive field productions, showed considerable variability in the contents of macronutrients and micronutrients, even though the plants were similarly cultivated. Wide ranges in nutrient levels in plants are quite common, as several environmental factors influence plants’ development and metabolic responses [23]. Considering this, our results for these species are consistent with the published data. No proximate analyses of the other species is available in the literature for comparison.

The comparison of chemical composition between green and control gnocchi highlighted, in particular, higher available and lower not available carbohydrate amounts in control gnocchi. As expected, the lack of vegetable ingredients influenced the proximate composition of control gnocchi, even though their recipes resembled those of traditional gnocchi.

### 3.3. Bioactive Compounds

#### 3.3.1. Carotenoids

**Figure 1:**
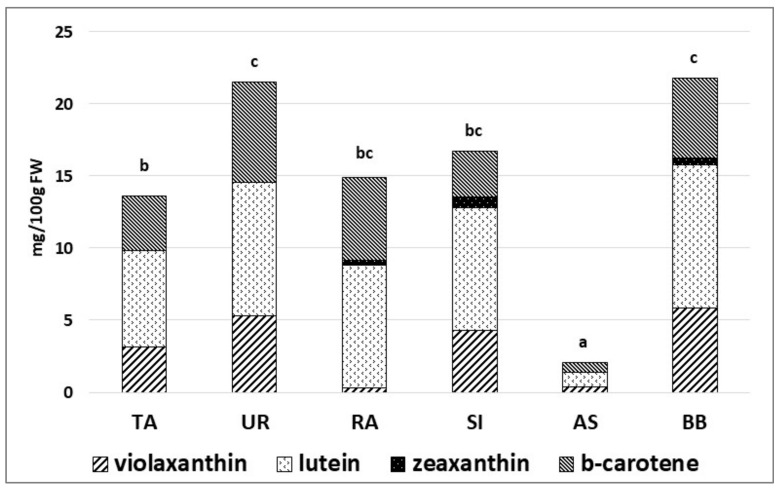
Total carotenoids (mg/100 g FW) in raw botanical species, analyzed in the HPLC. Bars of total carotenoid contents reporting different letters are significantly different (*p* < 0.05). *T. officinale* (TA), *U. dioica* (UR), *R. acetosa* (RA), *S. vulgaris* (SI), *A. dioicus* (AS), *B. bonus-henricus* (BB).

The variety in botanical species significantly affected total carotenoid contents in the raw samples, even though all species displayed the same type of carotenoid complex (Figure 1). The content of xanthophylls greatly exceeded that of carotenes; lutein represented approximately 50% of carotenoids identified, followed by β-carotene and violaxanthin. Only BB, SI, and RA contained traces of zeaxanthin. *B. bonus-henricus* and *U. dioica* showed the highest levels of carotenoids (more than 20 mg/100 g); *S. vulgaris*, *T. officinale*, and *R. acetosa* contained around 15 mg/100 g, while the level of carotenoids in *A. dioicus* was significantly lower than that of all the other wild species.

Total carotenoids were analyzed on raw and cooked samples using the spectrophotometric method to assess the effect of cooking (Table 6). Boiling the vegetables following traditional practice significantly affected total carotenoid levels in *S. vulgaris*, *B. bonus-henricus* (BB), and *A. dioicus* (AS). The increase in carotenoid content in AS should not be surprising. As a matter of fact, several studies have shown that they may increase after boiling [37,38]. This is attributed to the improved solvent extractability due to the impact of heat treatment on the food matrix, an effect that is related to food structure and carotenoid compound [39]. Nevertheless, the results confirmed the low content of carotenoid in AS found by using the HPLC method. The correlation between data of total carotenoids in raw samples for each gatherer (*n* = 14) determined by the two analytical methods was significant (r = 0.6781 and *p* < 0.001), although the spectrophotometric method underestimated the contents.

Although there are no dietary recommendation levels for carotenoids that are recognized by international agencies, optimal intakes of these compounds have been related to reduced risks of developing certain cancers, cardiovascular diseases, as well as bone, skin, and eye disorders and, more recently, brain-related diseases [40]. The dietary intake of carotenoids varies in the European population from 1 to 22 mg/day [41]. In Italy, the intake is around 10 mg/day, mainly as lycopene, β-carotene, and lutein/zeaxanthin [42]. Green leafy vegetables are a major source of these compounds in the human diet, for instance, the mean content of total carotenoids in spinach ranges from 9.0 to 12.7 mg/100 g FW [43]. The wild species that we investigated, with the only exception of AS, have higher levels of carotenoids compared to commercially available vegetables [40], and are an important dietary source of lutein (i.e., BB: 9.9 mg/100 g FW; UR: 9.3 mg/100 g FW) and violaxanthin (i.e., BB: 5.9 mg/100 g FW; UR 5.4 mg/100 g FW). Lutein, in particular, has attracted significant attention due to its beneficial effects on ocular diseases, such as age-related macular degeneration and diabetic retinopathy [44].

In this study, the carotenoid profiles detected in raw samples by HPLC are consistent with data of other works [45,46,47], although the total amounts of carotenoids could vary. Considering *U. dioica*, one of the most studied species, we found 21.6 mg of total carotenoids/100 g FW (or 138 mg/100 g dried weight, DW); other studies have reported similar [46], higher [34], or lower values [45,47]. The genotype, growth conditions, harvest and post-harvest treatments, and the analytical procedures might account for the differences [48].

Finally, some carotenoids, such as β-carotene, α-carotene, and β-cryptoxanthin are known as provitamin A. Based on the β-carotene results, the retinol equivalent contents in the wild plants sorted in descending order are: UR (1158 µg RE/100 g FW) > RA (967 µg RE/100 g FW) > BB (924 µg RE/100 g FW) > TA (633 µg RE/100 g FW) > SI (533 µg RE/100 g FW) > AS (117 µg RE/100 g FW). The consumption of one portion of 150 g of the wild species investigated herein (with AS as the only exception) might cover the daily need of vitamin A of an adult person (PRI of 700 µg RE/day, LARN 2014 [49]).

#### 3.3.2. Total Phenolic Contents

The contents of total phenolic compounds were determined on raw and cooked vegetable species; the results are reported in Table 6. Raw *R. acetosa* (RA) showed the highest content of phenols, while *U. dioica* (UR) showed the lowest content of phenols. The levels of the other raw vegetable species were included in a narrow range (288–372 mg GAE/100 g FW). Cooking significantly decreased the phenolic contents in plants; *S. vulgaris* (SI), *A. dioicus* (AS), and *B. bonus-henricus* (BB) were particularly susceptible to phenol degradation or leaching by boiling treatment. Decreases of total phenolic contents by boiling seems to be related to intensity of cooking treatment and type of vegetables [50].

Phenolic compounds are a complex class of plant secondary metabolites, widely distributed in vegetable foods. These substances have been studied mainly in relation to their antioxidant and anti-inflammatory properties, but recently, other mechanisms of action with beneficial health effects have been emerging, such as the modulation of gene expression and transcription factors. Long-term consumption of diets high in phenolics contributes to lower the risk of diseases related to oxidative or inflammatory imbalances, as well as of many chronic diseases [51]. The dietary intake of these compounds in Europe greatly varies either for quantity, from 600 to 1700 mg/day, or quality, depending on dietary patterns [52]. For some of the most common cultivated vegetables, the levels are as follows: spinach ~9–13 mg GAE/g DW, broccoli ~9–12 mg GAE/g DW, and kale ~16–19 mg GAE/g DW [50,53]. Total phenolics of raw wild plants analyzed in this study (when expressed on a dry basis), are between 9.2 (UR) and 39.8 (RA) mg GAE/g DW. These values are higher than in commercially available vegetables. A comparison with the literature data is possible for *U. dioica*; considering our results, many studies have found total phenolic contents to be similar [23,54,55,56] or higher [31,34,36,57] in leaves. In these latter works, the extraction phase was maximized by treatment at a high temperature (50 °C) or by sonication. Information on the total phenolic content can also be found for *S. vulgaris* from Spain. Morales Gómez reported lower contents in fresh leaves of bladder campion than the results reported in our study (62.38 mg GAE/100 g FW) [58,59]. While the preparation and analyses methods were comparable to ours, this variability could be easily explained taking into consideration the completely different pedoclimatic characteristics of the gathering area, as well as the different altitudes of the sites and the periods of harvesting. In wild nettle, Repajic et al. (2021) demonstrated that plant phenological stage and growing habitat significantly affected amounts of bioactive compounds [23].

#### 3.3.3. Mineral Contents

**Figure 2:**
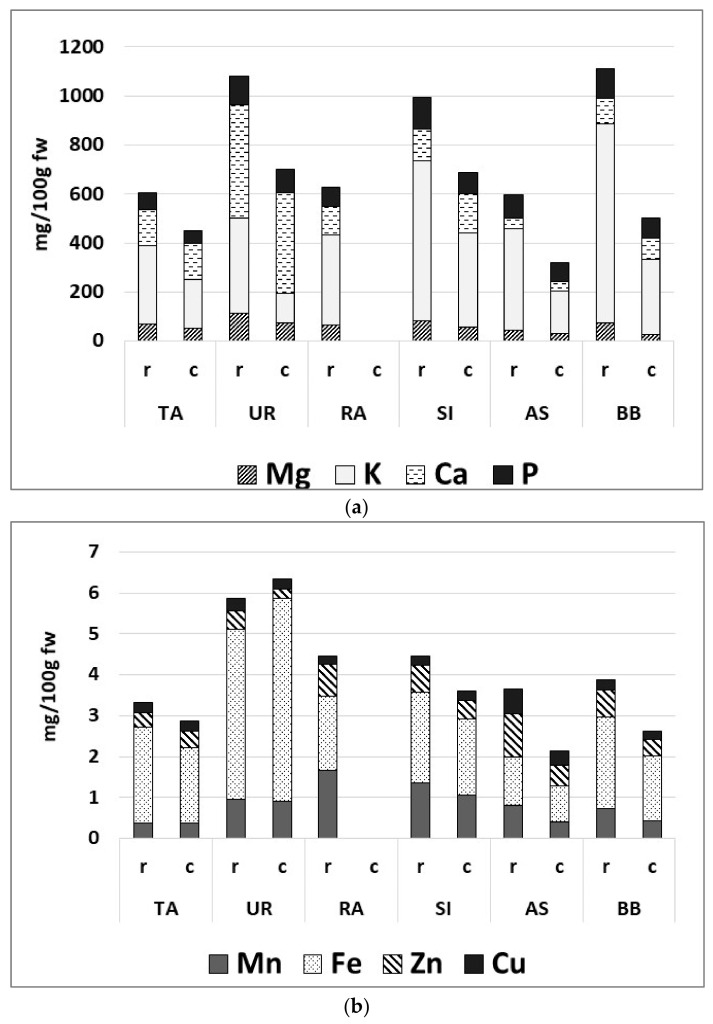
Contents of macro- (**a**) and micro- (**b**) elements in raw and cooked plants (mg/100 g FW). *T. officinale* (TA), *U. dioica* (UR), *R. acetosa* (RA), *S. vulgaris* (SI), *A. dioicus* (AS), *B. bonus-henricus* (BB). r, raw sample and c, cooked sample.

The wild plants displayed peculiar mineral contents (Figure 2a,b). UR was of special interest as a dietary source of minerals, in particular Fe, Ca, and Mg (0.9 ± 0.1, 409.6 ± 176.4, and 72.3 ± 27.6 mg/100 g FW, respectively). Raw RA had the highest level of Mn and Zn (79.0 ± 15.4 and 1.8 ± 0.0 mg/100 g FW), while in cooked plants, a high value of Mn was found in SI (1.4 ± 0.1 mg/100 g FW). AS contained relevant amounts of trace minerals, such as Zn and Cu (0.5 ± 0.3 and 0.3 ± 0.1 mg/100 g FW). Cooking influenced mineral levels differently, causing, for instance, a significantly decrease of K and Mg in the cooked samples, while the other minerals were essentially unaffected. The phosphorous contents ranged between 67 and 127 mg/100 g FW in the raw samples and between 51 and 96 mg/100 g FW in the cooked samples.

Among the wild plants examined in the present study, raw and boiled *U. dioica* stands out as a prominent dietary source of minerals; the consumption of a small portion of 50 g covers about one fifth of the daily requirements of Mg, Ca, and Fe for an adult [49]. These results confirm the literature data regarding wild *Urtica* spp. collected in other areas, such as the Mediterranean area [60]. However, it is important to note that the bioavailability of these elements may be lower in vegetable sources as opposed to animal sources due to the potential presence of anti-nutritional factors, such as oxalic acid [20,61,62,63]. Moreover, other than being able to reduce the bioavailability of minerals, high oxalic acid levels in plants could also be the cause for nephrolithiasis [60]. According to García-Herrera and Sánchez-Mata (2016), green leafy vegetables (i.e., such as some *Rumex* spp., *S. vulgaris*, and some *Taraxacum* spp.) should be avoided by people who suffer from kidney stones and other renal disorders despite their high content in Ca, due to the presence of high concentrations of this organic acid, and the preferable oxalic acid/Ca ratio in plant foods should be lower than 2.5 [60]. Furthermore, food processing, such as cooking, might alter, either by increasing or reducing, the influence of these anti-nutritional factors [64,65]. Therefore, ad hoc analyses are currently being planned in order to evaluate this aspect.

Finally, considering the ability of some wild species, such as some *Rumex* spp. and some *Taraxacum* spp., to hyperaccumulate heavy metals from the soil [66], further analyses regarding this issue might be interesting.

#### 3.3.4. Starch Digestibility in Gnocchi

**Figure 3:**
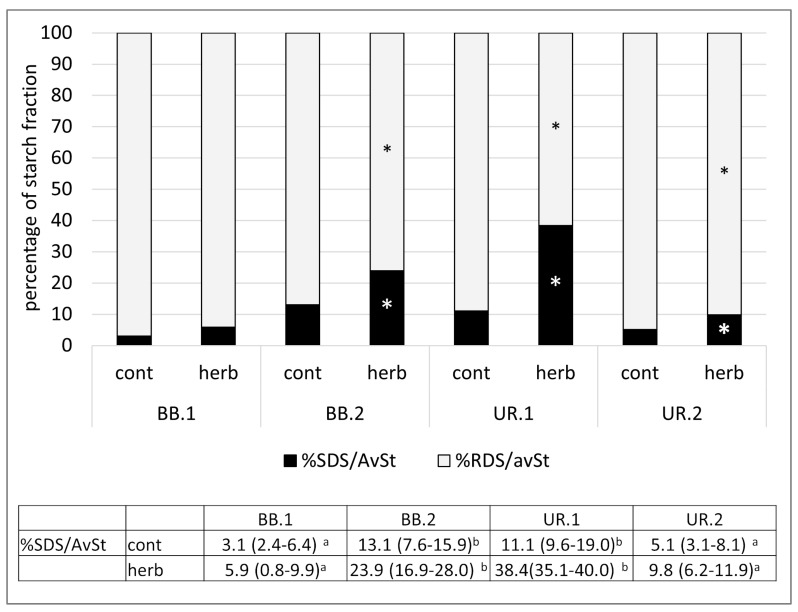
Percentage of slowly digestible starch (%SDS) and rapidly digestible starch (%RDS) on available starch in gnocchi with (herb) and without (cont) vegetable species, *B. bonus-henricus* (BB.1 and BB.2) and *U. dioica* (UR.1 and UR.2) * Significantly different from relative %SDS/AvST and %RDS/AvSt of control gnocchi (*p* < 0.05). In the table (results expressed as median (25–75° percentile)), it is reported the statistical analysis of the percentage of SDS/AvSt within the control or green gnocchi; data on the same row not sharing a common letter are significantly different (*p* < 0.05).

In vitro analyses of starch digestibility following the Englyst procedure has been recognized by the EFSA as a reliable method for estimating the impact of cereal product consumption on post-prandial glycemic responses. The following health claim on slowly digestible starch (SDS) has been approven by the European Food Safety Authority: “cereal products high in slowly digestible starch raise blood glucose concentrations less after a meal than cereal products low in slowly digestible starch”, together with the conditions for claim application [67]. In a previous study by Garsetti et al. (2005), rapidly digestible starch (RDS) was directly related to glycemic responses, whereas SDS was inversely correlated to insulin demand [68]. None of our samples of gnocchi with herbs achieved the conditions for the health claim, but the SDS fraction results were significantly higher in green gnocchi than in the corresponding control (*p* < 0.05) (Figure 3). These results, together with the lower contents of available starch in green gnocchi (Table 5), allow us to hypothesize that the consumption of one portion of green gnocchi might have a lower impact on postprandial glycemic responses than the control gnocchi. Focusing attention on the SDS fraction (the fraction of starch with positive impact on health), both the green gnocchi UR showed significantly higher percentages of SDS/AvSt compared to the corresponding control (*p* < 0.05), and the starch fraction in green gnocchi UR.1 was significantly higher than that in UR.2 (*p* < 0.05). This result is probably due to the more than double amount of *U. dioica* used as an ingredient in gnocchi in UR.1 (38.5%) vs. UR.2 (17%). As a matter of fact, several studies have suggested an inhibitory effect of phenolic compounds on starch digestion either by affecting the activities of α-amylase and/or amyloglucosidase, or by their binding interaction with the starch [69]. These actions might have both determined potentially retarded starch digestion (increasing SDS fraction), as found in our UR sample. Regarding gnocchi with BB, the amounts of herbs in the two gnocchi formulations were similar (11.7% in BB.2 vs. 14.9% in BB.1); however, SDS/AvSt in green gnocchi BB.2 was significantly higher than in BB.1 (*p* < 0.05). This result should be due to the different particle sizes of the herb used, because in the BB.2 gnocchi, herbs were blended with a mixer, while, in BB.1, the leaves were chopped with a knife. The difference in particle size could result in a difference in the matrix structure (or interaction between the components of BB and starch); thus, affecting the digestibility of starch in the BB.2 sample. In actual fact, proper food formulation and processing, aimed at hindering starch hydrolysis, are currently being investigated extensively with the purpose of reducing the glycemic impact of food consumption while preserving the sensory characteristic of food [70]. Finally, the in vitro method applied to investigate starch digestibility [32] was standardized to limit the influence of the food matrix. For example, it is known that some nutrients, such as lipid or dietary fiber, affect the physiological response to carbohydrates intake in vivo. However, these influences on starch accessibility are exerted by also involving the entire gastrointestinal system, while they are less determinant in the in vitro method.

## 4. Conclusions

The Alpine diet is an integral part of the Valmalenco territory. Along with the consumption of meat-based dishes, the Alpine diet includes a rich tradition of seasonal gathering and consumption of autochthonous plant species that are typically eaten fresh, especially during the months between April and June. They are consumed either cooked or raw and make up several of the dishes of the traditional Valmalenco cuisine. In some cases, the local population use preservation methods, such as freezing, in order to enable consumption of the plants even in months less conducive to gathering.

There is a lack of scientific data in the literature regarding the nutritional characteristics of autochthonous plant species gathered along the Alps. Our work has highlighted interesting results in this regard. For example, the level of carotenoids was found, on average, to be higher in wild plants gathered for this study as compared to in commercially available vegetables. Lutein levels are of particular interest, as well as the contents of some of the carotenes that are considered to be provitamin A; and thus, they contribute indirectly to the recommended daily intake of Vitamin A. Concerning total phenols, raw *R. acetosa* was the sample with the highest levels. Although cooking significantly affects the nutrient contents in plants, *R. acetosa* is consumed exclusively raw as a field snack throughout the territory. Therefore, lower phenolic contents due to cooking are not a factor for this specific plant. Furthermore, *U. dioica* seems to be a prominent dietary source of minerals, especially Mg, Ca, and Fe, confirming the literature data available on this species, although further investigations on the bioavailability of these elements are needed. Moreover, special attention should be paid to potential antinutritional factors or toxic compounds in this species, such as oxalic acid in *Rumex* spp., *Silene vulgaris*, and *Taraxacum* spp., or cyanogenetic glycosides in *Aruncus dioicus*.

Concerning the typical dish green gnocchi (either with wild spinach or nettles), the SDS fraction was found to be significantly increased compared to the matched control recipe. This result is of particular interest considering the inverse correlation of this fraction with insulin demand. Additionally, hand-chopped versus blended wild spinach resulted in a difference in the digestibility of starch, which may have been caused by the difference in matrix structures. This last result merits further investigation in a study aimed at examining the potential correlation between food formulation and its consumption’s glycemic impact.

However preliminary, the data, as presented in this study, already highlight the nutritional value of the plant species traditionally used in Valmalenco, which are such an important component of the territory diet. Furthermore, it is important not to neglect the relevance of these types of studies because, in addition to enriching the scientific literature, they also contribute to the protection and enhancement of the territory, its traditions, and its cultural and plant biodiversity.

## Figures and Tables

**Table 1:** Collection, processing, and transportation steps for raw and cooked samples of the six species.

Species Sample Code *Herbarium* Specimen Code	Information about Collection	Processing following Traditional Methods	Conservation and Transportation
*Aruncus dioicus* L. AS.1-Raw AS-21-001	**Part of the plant:** red-purplish young sprouts **Altitude:** 850–900 m a.s.l. **Time of collection:** 12:00 p.m. **Weather:** cloudy **T:** 10 °C **Humidity:** 42%	The sprouts are washed in running tap water, and then the last 3–4 cm of the stems are carefully peeled with a knife.	Put into a refrigerator at +4 °C, and then transported to the laboratory within 24 h, maintaining the chain cold.
*Aruncus dioicus* L. AS.1-Cooked AS-21-001	**Part of the plant:** red-purplish young sprouts **Altitude:** 850–900 m a.s.l. **Time of collection:** 12:00 p.m. **Weather:** cloudy **T:** 10 °C **Humidity:** 42%	The sprouts are washed in running tap water, and then 3–4 cm of the stem ends are carefully peeled with a knife. Then, they are patted dried with a clean tablecloth. **First boiling:** The stems are placed in a pot with cold water. When the water reaches the boiling point, they are left for 1–2 min. The water is thrown away. **Second boiling:** The blanched sprouts are put in already boiling water for 8 min. After draining, they are left to dry and cool.	Frozen at −20 °C, and then transported to the laboratory within 24 h, maintaining the chain cold.
*Aruncus dioicus* L. AS. 2-Raw AS-21-001	**Part of the plant:** red-purplish and red-greenish young sprouts **Altitude:** 1000 m a.s.l. **Time of collection:** 5:00 p.m. **Weather:** cloudy **T:** 11 °C **Humidity:** 45%	The remaining 3–4 cm of the stem ends are carefully peeled with a knife to remove the dirt. The plant is not washed with water.	Put at +4 °C in a fridge, and then transported to the laboratory within 24 h, maintaining the chain cold.
*Aruncus dioicus* L. AS.2-Cooked AS-21-001	**Part of the plant:** red-purplish and red-greenish young sprouts **Altitude:** 1000 m a.s.l. **Time of collection:** 5:00 p.m. **Weather:** cloudy **T:** 11 °C **Humidity:** 45%	The remaining 3–4 cm of the stem ends are carefully peeled with a knife to remove the dirt. Then, the plant is washed with water ahead of cooking. **First boiling:** The stems are placed in a pot with cold water. When the water reaches the boiling point, they are left for 2–3 min. Then, the water is thrown away. **Second boiling:** The blanched sprouts are put in already boiling water for 10 min. After draining, they are left to dry and cool.	Frozen at −20 °C, and then transported to the laboratory within 24 h, maintaining the chain cold.
*Blitum bonus-henricus* (L.) Rchb. BB.1-Raw BB-21-001	**Part of the plant:** Young leaves **Altitude:** 1600 m a.s.l. **Time of collection:** 4:30 p.m. **Weather:** sunny **T:** 13 °C **Humidity:** 30%	Washed in tap water and spin-dried.	Put at +4 °C in a fridge, and then transported to the laboratory within 24 h, maintaining the chain cold.
*Blitum bonus-henricus* (L.) Rchb. BB.1-Cooked BB-21-001	**Part of the plant:** Young leaves **Altitude:** 1600 m a.s.l. **Time of collection:** 4:30 p.m. **Weather:** sunny **T:** 13 °C **Humidity:** 30%	Washed in tap water. **Boiling:** The leaves are placed in a pot with cold water. When the water reaches the boiling point, they are left for 5 min and stirred from time to time. They are placed under cold tap water, spin-dried, and then manually wrung.	Frozen at −20 °C, and then transported to the laboratory within 24 h, maintaining the chain cold.
*Blitum bonus-henricus* (L.) Rchb. BB.2-Raw BB-21-001	**Part of the plant:** Young leaves **Altitude:** 1580 m a.s.l. **Time of collection:** 11:30 a.m. **Weather:** cloudy **T:** 9 °C **Humidity:** 60%	Washed in tap water and dried with a tablecloth.	Put at +4 °C in a fridge, and then transported to the laboratory within 24 h, maintaining the chain cold.
*Blitum bonus-henricus* (L.) Rchb. BB.2-Cooked BB-21-001	**Part of the plant:** Young leaves **Altitude:** 1580 m a.s.l. **Time of collection:** 11:30 a.m. **Weather:** cloudy **T:** 9 °C **Humidity:** 60%	Washed in tap water. **Boiling:** The leaves are placed in a pot with cold water. When the water reaches the boiling point, they are left for 15 min and stirred from time to time. Then, they are then manually wrung.	Frozen at −20 °C, and then transported to the laboratory within 24 h, maintaining the chain cold.
*Rumex acetosa* L. RA.1-Raw RA-21-001	**Part of the plant:** Leaves and young stems **Altitude:** 1300 m a.s.l. **Time of collection:** 10:00 a.m. **Weather:** cloudy **T:** 10 °C **Humidity:** 57%	None	Put at +4 °C in a fridge, and then transported to the laboratory within 24 h, maintaining the chain cold.
*Rumex acetosa* L. RA.2-Raw RA-21-001	**Part of the plant:** Leaves and young stems **Altitude:** 1300 m a.s.l. **Time of collection:** 11:00 a.m. **Weather:** cloudy **T:** 10 °C **Humidity:** 57%	None	Put at +4 °C in a fridge, and then transported to the laboratory within 24 h, maintaining the chain cold.
*Silene vulgaris* L. SI.1-Raw SI-21-001	**Part of the plant:** Young leaves **Altitude:** 1100 m a.s.l. **Time of collection:** 3:30 p.m. **Weather:** sunny **T:** 13 °C **Humidity:** 30%	Washed in tap water and spin-dried.	Put at +4 °C in a fridge, and then transported to the laboratory within 24 h, maintaining the chain cold.
*Silene vulgaris* L. SI.1-Cooked SI-21-001	**Part of the plant:** Young leaves **Altitude:** 1100 m a.s.l. **Time of collection:** 3:30 p.m. **Weather:** sunny **T:** 13 °C **Humidity:** 30%	Washed in tap water and spin-dried. **Boiling:** The leaves are placed in a pot with cold water. When the water reaches the boiling point, they are left for 5 min and stirred from time to time. They are spin-dried, and then manually wrung.	Frozen at −20 °C, and then transported to the laboratory within 24 h, maintaining the chain cold.
*Silene vulgaris* L. SI.2-Raw SI-21-001	**Part of the plant:** Young leaves **Altitude:** 1100 m a.s.l. **Time of collection:** 2:30 p.m. **Weather:** cloudy, light rain **T:** 10 °C **Humidity:** 57%	Washed in tap water and spin-dried.	Put at +4 °C in a fridge, and then transported to the laboratory within 24 h, maintaining the chain cold.
*Silene vulgaris* L. SI.2-Cooked SI-21-001	**Part of the plant:** Young leaves **Altitude:** 1100 m a.s.l. **Time of collection:** 2:30 p.m. **Weather:** cloudy, light rain **T:** 10 °C **Humidity:** 57%	Washed in tap water and spin-dried. **Boiling:** The leaves are placed in a pot with cold water. When the water reaches the boiling point, they are left for 5 min and stirred from time to time. They are placed under cold tap water, spin-dried, and then manually wrung.	Frozen at −20 °C, and then transported to the laboratory within 24 h, maintaining the chain cold.
*Taraxacum officinale* Weber TA.1a-Raw TA-21-001	**Part of the plant:** Leaves, adult plant **Altitude:** 1100 m a.s.l. **Time of collection:** 2:30 p.m. **Weather:** cloudy, light rain **T:** 10 °C **Humidity:** 57%	Washed in tap water and spin-dried.	Put at +4 °C in a fridge, and then transported to the laboratory within 24 h, maintaining the chain cold.
*Taraxacum officinale* Weber TA.1a-Cooked TA-21-001	**Part of the plant:** Leaves, adult plant **Altitude:** 1100 m a.s.l. **Time of collection:** 2:30 p.m. **Weather:** cloudy, light rain **T:** 10 °C **Humidity:** 57%	Washed in tap water and spin-dried. **Boiling:** The leaves are placed in a pot with cold water. When the water reaches the boiling point, they are left for 5 min and stirred from time to time. They are placed under cold tap water, spin-dried, and then manually wrung.	Frozen at −20 °C, and then transported to the laboratory within 24 h, maintaining the chain cold.
*Taraxacum officinale* Weber TA.1b-Raw TA-21-001	**Part of the plant:** Leaves, flowerheads, root mix, adult plant **Altitude:** 1100 m a.s.l. **Time of collection:** 2:30 p.m. **Weather:** cloudy, light rain **T:** 10 °C **Humidity:** 57%	The root is peeled in the field with a knife, until only a little piece remains. Then, the leaves and roots are washed in tap water and spin-dried. The flowerheads are patted with a tablecloth and the flowers are removed from the receptacle and added to the mix.	Put at +4 °C in a fridge, and then transported to the laboratory within 24 h, maintaining the chain cold.
*Taraxacum officinale* Weber TA.2a-Raw TA-21-001	**Part of the plant:** Leaves, adult plant **Altitude:** 1300 m a.s.l. **Time of collection:** 10:00 a.m. **Weather:** cloudy, light rain **T:** 10 °C **Humidity:** 57%	Washed in tap water and spin-dried.	Put at +4 °C in a fridge, and then transported to the laboratory within 24 h, maintaining the chain cold.
*Taraxacum officinale* Weber TA.2a-Cooked TA-21-001	**Part of the plant:** Leaves, adult plant **Altitude:** 1300 m a.s.l. **Time of collection:** 10:00 a.m. **Weather:** cloudy, light rain **T:** 10 °C **Humidity:** 57%	Washed in tap water and spin-dried. **Boiling:** The leaves are placed in a pot with cold water. When the water reaches the boiling point, they are left for 5 min and stirred from time to time. They are placed under cold tap water, spin-dried, and then manually wrung.	Frozen at −20 °C, and then transported to the laboratory within 24 h, maintaining the chain cold.
*Taraxacum officinale* Weber TA.2b-Raw TA-21-001	**Part of the plant:** Leaves, flowerheads, root mix, adult plant **Altitude:** 1300 m a.s.l. **Time of collection:** 10:00 a.m. **Weather:** cloudy, light rain **T:** 10 °C **Humidity:** 57%	The root is peeled in the field with a knife, until only a little piece remains. Then, the leaves and roots are washed in tap water and spin-dried. The flowerheads are patted with a tablecloth and the flowers are removed from the receptacle and added to the mix.	Put at +4 °C in a fridge, and then transported to the laboratory within 24 h, maintaining the chain cold.
*Urtica dioica* L. UR.1-Raw UR-21-001	**Part of the plant:** Leaves (adult plant) and tops **Altitude:** 1000 m a.s.l. **Time of collection:** 4:30 p.m. **Weather:** sunny **T:** 14 °C **Humidity:** 62%	Washed in tap water and spin-dried.	Put at +4 °C in a fridge, and then transported to the laboratory within 24 h, maintaining the chain cold.
*Urtica dioica* L. UR.1-Cooked UR-21-001	**Part of the plant:** Leaves (adult plant) and tops **Altitude:** 1000 m a.s.l. **Time of collection:** 4:30 p.m. **Weather:** sunny **T:** 14 °C **Humidity:** 62%	Washed in tap water and spin-dried. **Boiling:** The leaves are placed in a pot with cold water. When the water reaches the boiling point, they are left for 5 min and stirred from time to time. Then, they are manually wrung.	Frozen at −20 °C, and then transported to the laboratory within 24 h, maintaining the chain cold.
*Urtica dioica* L. UR.2-Raw UR-21-001	**Part of the plant:** Leaves (adult plant) and tops **Altitude:** 1100 m a.s.l. **Time of collection:** 10:00 a.m. **Weather:** sunny **T:** 21 °C **Humidity:** 53%	Washed in tap water and Na(HCO_3_), and then spin-dried.	Put at +4 °C in a fridge, and then transported to the laboratory within 24 h, maintaining the chain cold.
*Urtica dioica* L. UR.2-Cooked UR-21-001	**Part of the plant:** Leaves (adult plant) and tops **Altitude:** 1100 m a.s.l. **Time of collection:** 10:00 a.m. **Weather:** sunny **T:** 21 °C **Humidity:** 53%	Washed in tap water and Na(HCO_3_), and then spin-dried. **Boiling:** The leaves are placed in a pot with cold water. When the water reaches the boiling point, they are left for 5 min and stirred from time to time. Then, they are manually wrung.	Frozen at −20 °C, and then transported to the laboratory within 24 h, maintaining the chain cold.

**Table 2:** Traditional green gnocchi recipes followed during the study.

Species Code	Information about Collection	Processing and Traditional Recipes for the Preparation of Gnocchi	Conservation and Transportation
*Blitum bonus-henricus* L. BB.1-Gnocchi	**Part of the plant:** Young leaves **Altitude:** 1700 m a.s.l. **Time of collection:** 4:30 p.m. **Weather:** sunny **T:** 13 °C **Humidity:** 30%	Washed in tap water and spin-dried. **Recipe for 100 g of gnocchi dough:** - 51.4 g of refined wheat flour - 24.9 g of water - **14.9 g of fresh wild spinach leaves** - 7 g of stale bread - 2 g of extra virgin olive oil - A pinch of salt The leaves are placed in a pot with cold water. When the water reaches the boiling point, the leaves are left for 5 min and stirred from time to time. Then, the leaves are placed under cold tap water, spin-dried, and manually wrung. The leaves are then thinly chopped with a knife. The bread is soaked in water and smashed. All the ingredients are mixed until a soft dough is obtained. Then, the dough is divided using a spoon and the gnocchi are directly blanched in salted boiling water for 5 min.	Frozen at −20 °C, and then transported to the laboratory within 24 h, maintaining the chain cold.
*Blitum bonus-henricus* L. BB.2-Gnocchi	**Part of the plant:** leaves, young stems, flowering tops **Altitude:** 1280 m a.s.l. **Time of collection:** 9:30 a.m. **Weather:** cloudy **T:** 9 °C **Humidity:** 60%	Washed in tap water. **Recipe for 100 g of gnocchi dough:** - 50.5 g of white flour - 26 g of water - **11.7 g of fresh wild spinach** - 6 g of stale bread - 6 g of eggs - A pinch of salt The leaves are placed in boiling water for 15 min, and then, lightly manually wrung and blended with the immersion mixer.The bread is grated. All the ingredients are mixed until a soft dough is obtained. Then, the dough is divided using a spoon and the gnocchi are directly blanched in salted boiling water for 5 min.	Frozen at −20 °C, and then transported to the laboratory within 24 h, maintaining the chain cold.
*Urtica dioica* L. UR.1-Gnocchi	**Part of the plant:** Leaves and tops **Altitude:** 1000 m a.s.l. **Time of collection:** 3:30 p.m. **Weather:** sunny **T:** 14 °C **Humidity:** 62%	Washed in tap water and spin-dried. The leaves are placed in a pot with cold water. When the water reaches the boiling point, they are left for 5 min and stirred from time to time. Manually wrung. **Recipe for 100 g of gnocchi dough:** - 48 g of white flour - **38.5 g of cooked nettles** - 10 g of the nettle cooking water - 3.5 g of egg white - A pinch of salt The cooked leaves are blended with the immersion mixer with their cooking water. All the ingredients are gently mixed till a soft dough is obtained. Then, the dough is divided using a spoon and the gnocchi are directly blanched in salted boiling water for 5 min.	Frozen at −20 °C, and then transported to the laboratory within 24 h, maintaining the chain cold.
*Urtica dioica* L. UR.2-Gnocchi	**Part of the plant:** Adult tops **Altitude:** 1100 m a.s.l. **Time of collection:** 10:00 a.m. **Weather:** sunny **T:** 21 °C **Humidity:** 53%	Washed in tap water and Na(HCO_3_) and spin-dried. The leaves are placed in a pot with cold water. When the water reaches the boiling point, they are left for 5 min and stirred from time to time. Manually wrung. **Recipe for 100 g of gnocchi dough:** - 30 g of water - 29 g of white flour - **17 g of cooked nettles** - 15 g of whole milk - 11.5 g of eggs - 2 g of parmesan - A pinch of salt - A pinch of pepper - A pinch of nutmeg The leaves are thinly chopped with a knife. All the ingredients are gently mixed, by carefully avoiding lumps, till a soft dough is obtained. Then, the dough is divided using a spoon and the gnocchi are directly blanched in salted boiling water for 5 min.	Frozen at −20 °C, and then transported to the laboratory within 24 h, maintaining the chain cold.

**Table 3:** Plant species mentioned in Valmalenco at least 50 times in the food sector, considering only current and first-person use.

Species	Part of the Plant	Main Traditional Use	No. of Citations
*Vaccinium myrtillus* L.	Fruits	Fresh fruits/Jams	709
*Rubus idaeus* L.	Fruits	Fresh fruits/Jams	510
*Taraxacum officinale* Weber	Leaves/young leaves/flowers/hypogeal parts	Salads, cooked vegetable side	453
*Fragaria vesca* L.	False fruits	Fresh fruits/Jams	386
*Vaccinium vitis-idaea* L.	Fruits	Fresh fruits/Jams	376
*Sambucus nigra* L.	Fruits	Fresh fruits/Jams	341
*Artemisia genipi* Weber ex Stechm.	Aerial parts	Liquors	300
*Urtica dioica* L.	Young leaves	Cooked vegetable side, gnocchi, risottos	282
*Thymus* spp.	Leaves	Flavoring	274
*Juniperus communis* L.	Galbula	Flavoring	270
*Achillea moschata* Wulfen	Aerial parts	Liquors	249
*Rosa canina* L.	False fruits	Fresh fruits/Jams	216
*Rubus ulmifolius* Schott	Fruits	Fresh fruits/Jams	209
*Blitum bonus-henricus* L.	Young leaves	Cooked vegetable side, gnocchi	203
*Gentiana lutea* L.	Hypogeal parts	Liquors, grappa	191
*Aruncus dioicus* (Walter) Fernald	Young sprouts/shoots	Cooked vegetable side, risottos	132
*Silene vulgaris* (Moench) Garcke	Young leaves	Cooked vegetable side, soups	111
*Malva sylvestris* L	Leaves	Soups	76
*Humulus lupulus* L	Young sprouts/shoots	Cooked vegetable side, risottos	76
*Ribes rubrum* L.	Fruits	Fresh fruits/Jams	66
*Salvia officinalis* L.	Leaves	Flavoring	61
*Prunus spinosa* L.	Fruits	Fresh fruits/Jams	60
*Pinus mugo* L.	Pinecones	Liquors, grappa	56
*Rumex acetosa* L.	Leaves/young stems	Raw snack	54

**Table 4:** Chemical composition of raw vegetable species.

	TA	UR	RA	SI	AS	BB
Moisture	88.0 (86.8–89.0) ^bc^	84.5 (83.1–85.6) ^a^	86.0 (85.8–86.4) ^ab^	86.3 (86.0–86.9) ^b^	89.2 (89.0–89.5) ^c^	87.2 (85.8–88.8) ^bc^
Protein	2.3 (1.9–2.7) ^a^	3.9±1.2 ^bc^	3.4 (3.1–4.8) ^b^	4.2 (4.1–4.2) ^bc^	3.2 (2.9–3.5) ^b^	5.0 (4.4–5.5) ^c^
Lipid	0.4 (0.3–0.5) ^ab^	0.3 (0.3–0.3) ^a^	0.9 (0.8–0.9) ^c^	0.6 (0.6–0.6) ^bc^	0.7 (0.7–0.7) ^c^	0.4 (0.4–0.4) ^ab^
CHO avail	2.3 (2.0–2.7) ^cd^	1.9 (1.7–2.0) ^bc^	2.5 (2.3–2.7) ^d^	1.6 (1.2–2.1) ^b^	1.2 (1.0–1.4) ^ab^	0.8 (0.7–0.8) ^a^
Ash	0.9 (0.8–1.0) ^a^	1.9 (1.7–2.1) ^b^	0.9 (0.8–1.0) ^a^	2.0 (1.8–2.1) ^b^	0.8 (0.8–0.8) ^a^	1.7 (1.5–1.8) ^b^
CHO not-avail *	6.2	7.6	6.3	5.4	4.8	5.0

Data are reported as g/100 g fresh weight (FW) (median (25–75° percentile)). CHO avail, available carbohydrates (free sugars and starch); CHO not-avail, not available carbohydrates. * Data are estimated as difference to 100 g. Within the same row, data not sharing common letters are significantly different (*p* < 0.05). *T. officinale* (TA), *U. dioica* (UR), *R. acetosa* (RA), *S. vulgaris* (SI), *A. dioicus* (AS), *B. bonus-henricus* (BB).

**Table 5:** Chemical composition of gnocchi.

With Herb	Gnocchi BB.1	Gnocchi BB.2	Gnocchi UR.1	Gnocchi UR.2
Moisture	60.4 (58.7–62.1) ^ab^	61.4 (61.2–61.5) ^bc^	56.6 (55.8–57.4) ^a^	68.9 (66.9–70.9) ^c^
Protein	6.1 (6.0–6.1) ^c^	5.6 (5.4–5.7) ^bc^	5.0 (4.9–5.0) ^a^	5.3(5.1–5.4) ^ab^
Lipid	2.1 (2.0–2.1) ^b^	1.2 (1.1–1.3) ^ab^	1.2 (1.1–1.2) ^a^	1.1 (1.0–1.1) ^a^
CHO avail	22.0 (21.7–22.3) ^b^	21.8 (20.9–22.4) ^b^	19.6 (18–20.9) ^ab^	13.3 (11.4–13.5) ^a^
Ash	0.8 (0.7–0.9) ^a^	0.9 (0.9–1.0) ^ab^	1.0 (1.0–1.1) ^bc^	1.1 (1.1–1.2) ^c^
CHO not-avail *	8.7	8.9	16.8	11.2
Resistant starch	1.1 ± 0.1 ^ab^	1.2 ± 0.1 ^b^	1.0 ± 0.0 ^a^	1.0 ± 0.1 ^a^
**Control**	**Gnocchi BB.1 C**	**Gnocchi BB.2 C**	**Gnocchi UR.1 C**	**Gnocchi UR.2 C**
Moisture	57.7 (57.6–57.7) ^b^ *	56.1 (55.7–56.5) ^a^ *	56.9 (56.5–57.3) ^ab^	56.5 (56.2–56.7) ^a^ *
Protein	4.8 (4.7–4.8) ^a^ *	6.0 (5.9–6.0) ^bc^ *	5.5 (5.4–5.6) ^ab^ *	10.0 (9.6–10.3) ^c^ *
Lipid	2.7 (2.7–2.8) ^bc^ *	1.7 (1.6–1.8) ^ab^ *	0.7 (0.7–0.7) ^a^ *	5.8 (5.7–5.8) ^c^ *
CHO avail	28.9 (29.4–29.5) ^ab^ *	29.4 (28.5–29.8) ^b^ *	32.0 (29.3–34.3) ^b^ *	21.6 (20.6–22.2) ^a^ *
Ash	0.8 (0.7–0.8) ^ab^	1.4 (1.1–1.8) ^c^ *	0.5 (0.5–0.6) ^a^ *	1.1 (1.0–1.1) ^bc^
CHO not-avail *	4.9	5.7	3.2	5.3
Resistant starch	1.2 ± 0.1 ^b^	1.1 ± 0.1 ^ab^	1.2 ± 0.1 ^b^ *	0.7 ± 0.1 ^a^ *

Data are reported as g/100 g fresh weight (FW) (median (25–75° percentile)). CHO avail, available carbohydrates (free sugars and starch); CHO not-avail, not-available carbohydrates. * Data are estimated as difference to 100 g. Gnocchi UR.1 and UR.2: gnocchi with *U. dioica*; Gnocchi UR.1 C and UR.2 C: gnocchi control matched with UR.1 and UR.2 ones; Gnocchi BB.1 and BB.2: gnocchi with *B. bonus-henricus*; Gnocchi BB.1 C and BB.2 C: gnocchi control matched with BB.1 and BB.2 ones. Data on the same row not sharing common letters are significantly different (*p* < 0.05). * Means significantly different from relative green gnocchi (*p* < 0.05).

**Table 6:** Total phenol (mg GAE/100 g FW) and carotenoid (mg/100 g FW) contents analyzed by using spectrophotometric methods, in wild raw and cooked samples.

	Total Phenols		Total Carotenoids	
	Raw Sample	Cooked Sample	Raw Sample	Cooked Sample
**TA**	264.3(129.5–456.9) ^b^	210.7(165.3–270.4) ^b^	9.9 (9.5–11.2) ^b^	11.8 (8.9–15.4) ^c^
**UR**	142.7(61.7–227.5) ^a^	76.3(40–118.0) ^a^	11.1 (7.5–16) ^bc^	15.1 (9.5–21.5) ^c^
**RA**	552.3(524.4–585.1) ^d^		15.1 (13.9–16.4) ^c^	
**SI**	374.5 (367.0–375.8) ^cd^	260.9 (251.7–296.5) ^b^ *	11.6 (10.2–12.3) ^bc^	8.8 (8.4–9.4) ^bc^ *
**AS**	305.7(280.2318.0) ^bc^	207.5(155.0–278.1) ^b^ *	1.2 (1.0–1.4) ^a^	1.8 (1.6–2.1) ^a^ *
**BB**	295.4(255.0–333.1) ^bc^	81.8 (53.8–110.7) ^ab^ *	10.1 (9.8–11.0) ^b^	6.1 (5.3–7.9) ^ab^ *

Data (median (25–75° percentile)) not sharing common letters within the same column of raw or cooked samples are significantly different (*p* < 0.05). * Significantly different from raw sample (*p* < 0.005). *T. officinale* (TA), *U. dioica* (UR), *R. acetosa* (RA); *S. vulgaris* (SI), *A. dioicus* (AS), *B. bonus-henricus* (BB).

## Data Availability

This paper contains all data concerning the nutritional analyses on plants. Complete data on interviews are unavailable due to privacy restrictions.
